# Bell Jar: A Semiautomated Registration and Cell Counting Tool for Mouse Neurohistology Analysis

**DOI:** 10.1523/ENEURO.0036-23.2025

**Published:** 2025-02-03

**Authors:** Alec L. R. Soronow, Matthew W. Jacobs, Richard G. Dickson, Euiseok J. Kim

**Affiliations:** ^1^Department of Molecular, Cell, and Developmental Biology, University of California, Santa Cruz, California 95064; ^2^Institute for the Biology of Stem Cells, University of California, Santa Cruz, California 95064

**Keywords:** alignment, atlas registration, brain mapping, cell detection, cell quantification, neuroanatomy

## Abstract

For comprehensive anatomical analysis of a mouse brain, accurate and efficient registration of the experimental brain samples to a reference atlas is necessary. Here, we introduce Bell Jar, a semiautomated solution that can align and annotate tissue sections with anatomical structures from a reference atlas as well as detect fluorescent signals with cellular resolution (e.g., cell bodies or nuclei). Bell Jar utilizes Mattes mutual information-directed B-spline transformations to achieve precise alignments, even with damaged sample tissues. While user input remains a requirement for fine-tuning section matches, the platform streamlines the process, aiding rapid analyses in high-throughput neuroanatomy studies. As a standalone desktop application with a user-friendly interface, Bell Jar’s performance, which surpasses traditional manual and existing automated methods, can improve the reproducibility and throughput of histological analyses.

## Significance Statement

Studies elucidating the anatomical organization of the brain are becoming increasingly complex in neuroscience. Thus, need for tools that can sufficiently handle increasing complexity and throughput of experiments is also increasing. While many options currently exist for performing these analyses, most do not provide an all-in-one guided workflow. Due to this smattering of tools, many labs must develop custom solutions. However, only some researchers have the programming expertise to troubleshoot and combine existing platforms to achieve their desired results. This is why we created Bell Jar, an open-source, multi-platform semiautomated toolkit that is usable by researchers of all skill levels. Bell Jar’s semiautomated nature and ease of installation allow any lab to process its experimental data quickly.

## Introduction

Due to the technological advances of microscopy and computer systems, large image datasets are becoming increasingly common among neuroscience researchers (Oh et al., 2014; Zingg et al., 2014; Kim et al., 2017; Landhuis, 2017; Harris et al., 2019), and there is a need to automate the analysis of this data. There can be hundreds of high-resolution brain tissue sections for analysis of whole-brain histology experiments with even a few animals (Oh et al., 2014; Zingg et al., 2014; Harris et al., 2019). Researchers must then interpret results manually to analyze experimental signals (e.g., count labeled cells) and demarcate anatomical regions (e.g., draw borders between different structures) to interpret results for each section. Manual annotation across large datasets is time-consuming and prone to interrater bias (Yates et al., 2019; Sadeghi et al., 2023). This bias is introduced in the traditional application of region boundaries by an expert with the aid of a paper atlas (Franklin and Paxinos, 2008). Each expert rater has to make a personal determination based on cues in the tissue section, such as cytoarchitecture or landmark structures. However, while these heuristics work for ideal tissue sections, their application becomes difficult for damaged or irregular tissue sections.

With these advances in brain imaging and the collection of large datasets, digital reference atlases have also become increasingly detailed and accessible. The Allen Brain Common Coordinate Framework (CCF; Wang et al., 2020) provides high-resolution atlas volumes and corresponding region annotations (including a coordinate system and structure ontologies) representing the average adult mouse brain. A computer program can be created to align an experimental tissue section into the reference atlas with a high-resolution digital intermediate tissue section as a guide (Xiong et al., 2018; Yates et al., 2019; Lauridsen et al., 2022), assuming they share key anatomical features. Using the resulting alignment, the exact annotations of the digital tissue section can be applied to determine the regions in the experimental tissue. These boundaries allow for further signal analysis within specific regions using additional software.

Several programs have been developed for alignment and signal detection (Xiong et al., 2018; Yates et al., 2019; Lauridsen et al., 2022; Sadeghi et al., 2023) and have seen significant usage throughout the neuroscience community, but they face limitations in their ability to align experimental tissues. While implementation details vary, they use the same principle discussed above: each performs some user-defined affine (Chiaruttini, 2019; Yates et al., 2019; Lauridsen et al., 2022) or user-defined spline-based transformation (Chiaruttini, 2019) of the digital atlas section to match the experimental tissue section. Affine transformations cannot accurately represent the extensive deformations arising from differences in the mounting and sectioning of individual experiments. This is due to their inability to represent complex elastic deformations. Tools that feature manual landmark-based deformable transformations (user-defined spline-transformations) of alignments address this issue. Still, the added time to manually tune hundreds of sections this way limits their utility. In some cases, this is a semiautomated user-driven process (Yates et al., 2019; Lauridsen et al., 2022). In others, it is automated based on deep learning or other heuristics (Carey et al., 2023; Sadeghi et al., 2023). Despite their efficacy, these tools still achieve limited accuracy on challenging tissues and cannot overcome complex deformations. Many of them also require 3D volumetric data from two-photon or lightsheet microscopy (Niedworok et al., 2016; Tyson et al., 2022), which may be inaccessible or nonapplicable to some neuroscience labs. Additionally, these tools are built on platforms that are consistently difficult to maintain and execute across platforms or as scripts in other software. Some of them are even completely defunct, paid use only, or not publicly available (Kopec et al., 2011; Mendez et al., 2018; Eastwood et al., 2019).

To address these limitations, we created Bell Jar: a standalone cross-platform desktop software which combines the ability to automatically register experimental tissues to the Allen Brain CCF and to quantify experimental signals based on said alignment, with minimal requirement for computational expertise. Bell Jar’s user interface (UI) and backend were built on the Electron.js framework (https://www.electronjs.org) to leverage web applications’ ease of development, powerful frameworks, and cross-platform compatibility. Bell Jar’s tools are written in Python as individual scripts. The software hosts its own standalone Python installation, keeping its dependencies separate from the host installations. These features let users download and start using the software with minimal configuration. Bell Jar provides various tools for histology analysis, with its functionality divided into independent tools: alignment, detection, and counting.

## Materials and Methods

### Experimental model details

GENSAT BAC transgenic SepW1-Cre NP39 and Tlx3-Cre PL56 mouse lines have been previously described (Gerfen et al., 2013). Both male and female mice were used. All animal procedures were performed in accordance with the University of California, Santa Cruz animal care committee’s regulations.

### Method details

#### Virus preparation

All AAVs and EnvA + RV*dG* were produced by the Salk Viral Core GT3: AAVretro-nef-lox66/71-tTA (1.77X10^12^ GC/ml), AAV8-TRE-DIO-oG-WPRE (5.92 × 10^12^ GC/ml), AAV8-TRE-DIO-eGFP-T2A-TVA (7.00 × 10^13 ^GC/ml), AAV8-DIO-TVA66T-2A-eGFP-2A-oG (5.06 × 10^13 ^GC/ml), and EnvA + RV*dG*-mCherry [1.07 × 10^9^ infectious unit (IU)/ml].

#### Animal surgery for virus injection

For rabies tracing experiments, SepW1-Cre NP39 or Tlx3-Cre PL56 mice received AAV helper injections at postnatal day (P)80-P100. Mice were anaesthetized with 100 mg/kg of ketamine and 10 mg/kg of xylazine cocktail via intraperitoneal injections and mounted in a stereotax (RWD instruments) for surgery and stereotaxic injections. In total, 50 nl of AAVretro-nef-lox66/71-tTA was injected into the center of medial secondary visual cortex (V2M), using the following coordinates: 1.8 mm caudal, 1.6 mm lateral relative to lambda, and 0.5–0.7 mm ventral from the pia. A 50 nl mixture of AAV8-TRE-DIO-oG-WPRE and AAV8-TRE-DIO-eGFP-T2A-TVA or a 50 nl AAV8-DIO-TVA66T-2A-eGFP-2A-oG was injected into the center of lateral secondary visual cortex (V2L), using the following coordinates: 0.7 mm rostral, 3.65 mm lateral relative to lambda, and 0.5–0.7 mm ventral from the pia. We injected AAVs using air pressure by 1 ml syringe with 18 G tubing adaptor and tubing. To prevent virus backflow, the pipette was left in the brain for 5–10 min after completion of injection. Two or three weeks after AAV helper injection, 100–200 nl of EnvA + RV*dG*-mCherry was injected into the same site in V2L using 1 ml syringe-mediated air pressure. Mice were housed for 7 d to allow for trans-synaptic rabies spread and fluorescent protein expression.

#### Histology and image analysis

Brains were harvested after trans-cardiac perfusion using phosphate-buffered saline (PBS) followed by 4% paraformaldehyde (PFA). Brains were dissected out from skulls and post-fixed with 2% PFA and 15% sucrose in PBS at 4°C overnight and then immersed in 30% sucrose in PBS at 4°C before sectioning. Using a freezing microtome, 50–100 µm coronal brain sections were cut and stored in PBS with 0.01% sodium azide at 4°C. To enhance eGFP and dsRed signals, free-floating sections were incubated at 4°C for 16–48 h with goat anti-GFP (1:1,000; Rockland 600-101-215) and rabbit anti-dsRed (1:500; Clontech 632496) primary antibodies in PBS/0.5% normal donkey serum/0.1% Triton X-100, followed by the appropriate secondary antibodies conjugated with Alexa 488 or 568 (1:500; Invitrogen A-11055 or A-10042). Sections were counterstained with 10 μM DAPI (4′,6-diamidino-2-phenylindole) in PBS for 30 min to visualize cell nuclei. Immunostained tissue sections were mounted on slides with polyvinyl alcohol mounting medium containing DABCO and allowed to air-dry overnight.

All sections were scanned with a 10× objective on a Zeiss Axio Imager Z2 Widefield Microscope. Subsequent image files were processed and analyzed by Bell Jar, ilastik, or NIH ImageJ (Fiji).

#### Statistical methods

Kruskal–Wallis one-way analysis from SciPy (Virtanen et al., 2020) of variance was used to analyze the significance of the difference between the alignment methods’ fit metrics, followed by post hoc Dunn to find statistical significances. Pearson’s product-moment correlation coefficient was used to calculate the correlation and significance for the various counting methods to manual data.

#### Hardware specifications

We performed all network training on a Windows 11 machine with an AMD Ryzen 9 7950X processor and an Nvidia RTX A5000 GPU. The minimum specifications we require for running Bell Jar are the following: an 8th generation Intel or Ryzen 5 (2017 onward) processor, a GTX 1060 (6 GB) GPU, and at least 2 GB of free hard drive space and network connectivity during the initial installation. The minimum GPU memory required for Bell Jar is 4 GB, but we recommend at least 6 GB and CUDA support for optimal performance.

#### Analysis of in situ hybridization signals

All images were collected from the Allen Brain CCF (Wang et al., 2020) transgenic characterization dataset via their public application programming interface. The experiments were individually aligned using Bell Jar’s alignment workflow. Images were first inverted and then converted to 8 bit grayscale, and in situ hybridization (ISH) signals were thresholded at a constant value (125) across all experiments to create binary masks of signal in each section. The number of thresholded pixels in each brain region was then quantified using the Bell Jar alignments.

#### Model generation and training

All YoloV8 (Jocher et al., 2023) models were trained using the large model weights for 200 epochs and with all recommended default hyperparameters. The predictor ResNet-101 (He et al., 2016) architecture was based on the architecture described in the original work with some key differences. Inputs to the network first undergo Sobel filtering to enhance the edges of anatomical features. The network’s final output dense layer is also modified to produce three normalized scalars representing our *x*-cut angle, *y*-cut angle, and anterior-posterior position. We normalized these values to aid training and generalization. Normalization of the vectors was done by assuming −10° to be the minimum cut angle in either dimension and 10° to be the maximum cut angle in either dimension. Likewise, for the anterior-posterior position, we based this off the indices of the digitally coronally sectioned Allen Mouse Brain Reference Atlas (Wang et al., 2020) 10 μm Nissl volume, with 0 being the minimum position and 1,324 being the maximum.

#### Composite transformation routine for alignment

The composite transformation routine aimed at aligning tissue sections to a reference atlas through a two-stage registration process, incorporating both affine and B-spline transformations. This process is facilitated by the SimpleITK framework (Lowekamp et al., 2013; Avants et al., 2014), a toolkit for medical image processing. Initially, the fixed (tissue section) and moving (reference atlas) images undergo padding, extended by a uniform margin of 50 pixels on all sides, to mitigate boundary effects during registration. Subsequently, these images are cast to a 32 bit floating-point format to ensure numerical precision in subsequent computations. Then, histogram matching is performed to give the reference atlas the same intensity distribution as our sample. The function employs the SimpleITK framework to adjust the intensity distribution of a moving image to match that of a fixed image, a process known as histogram matching. This is achieved through the “HistogramMatchingImageFilter” function, which modifies the pixel values of the moving image such that its histogram aligns with the histogram of the fixed image. The filter is configured to use 1,024 histogram levels, ensuring a detailed representation of intensity distributions, and it matches the histograms based on 10 equally spaced intensity values (match points) that are representative of the entire intensity range. Additionally, by activating the “ThresholdAtMeanIntensityOn” option, the algorithm modifies the intensity values of the moving image only above its mean intensity, thereby preserving the lower intensity range. Afterward we proceed with the first stage of registration.

#### Affine registration

The first stage involves an affine registration, where the moving image is aligned to the fixed image through an affine transformation. This is initialized using the “CenteredTransformInitializer” function which aligns the centers of the two images and estimates an initial transformation matrix. The affine registration employs the Mattes Mutual Information (Mattes et al., 2001) metric, a statistical similarity measure, with 32 histogram bins to guide the optimization process. Optimization is performed using gradient descent, with a learning rate of 0.01, over 300 iterations, or until convergence is reached, as indicated by a minimum value change of 1 × 10^−8^ over a 20-iteration window. Physical shifts determine the optimizer scales to ensure uniform step sizes across dimensions. A multiresolution strategy is employed by setting shrink factors and smoothing sigmas per level to [4, 2, 1] and [2, 1, 0], respectively, to enhance convergence. The transformation is interpolated using a linear method.

The affine transformation, denoted as *T*_affine_ (x), modifies the moving image to align with the fixed image based on translation, rotation, scaling, and shearing adjustments, formalized as follows:

$$T_{\mathrm{affine}}(x)=Ax+b,$$

where *A* is a linear transformation matrix and *b* is a translation vector applied to coordinates *x* of the moving image. When the affine transformation is found, we apply it to the fixed image and begin the second stage with that transformed image.

#### B-spline registration

The second stage of registration is conducted using a B-spline transformation. This is initialized over a specified mesh size (4 × 4), determined by a uniform grid with dimensions proportional to the image size, to introduce local deformations for finer alignment. This stage also utilizes gradient descent optimization with the same parameters as the affine stage, with the exception of now using neighborhood correlation as its fit metric. It also runs over 300 iterations to refine the alignment based on local deformations within the control points defined by the mesh. The B-spline transformation, *T*_B-spline_, allows for elastic deformation of the moving image to fit the fixed image more accurately, represented as follows:

$$T_{B-\mathrm{spline}}(x)=\overset{N}{\underset{i}{\sum }}\;P_{i}B_{i}(x),$$

where *P_i_* are the control points, *B_i_*(*x*) are the B-spline basis functions, and *N* is the number of control points. This method offers a flexible, high-degree-of-freedom approach to accommodate complex anatomical variations.

The final output is a composite transformation, combining the affine and B-spline transformations. This composite ensures that the initial global alignment provided by the affine transformation is refined through local adjustments via the B-spline transformation. The resulting composite transformation is applied to the moving image, achieving an aligned reference atlas section with respect to the fixed tissue image. The same transform is also applied to the reference atlas annotations so the labels may be applied to the tissue section.

### Running the Bell Jar workflow

Step-by-step installation and implementation can be also found on the GitHub page (https://github.com/asoronow/belljar/blob/main/docs/belljar_guide.pdf) or in Extended Data 1. All new users are advised to strictly adhere to the Bell Jar guide and follow the outlined steps for processing sample datasets before working with their own unique data.

## Results

**Figure 1.**
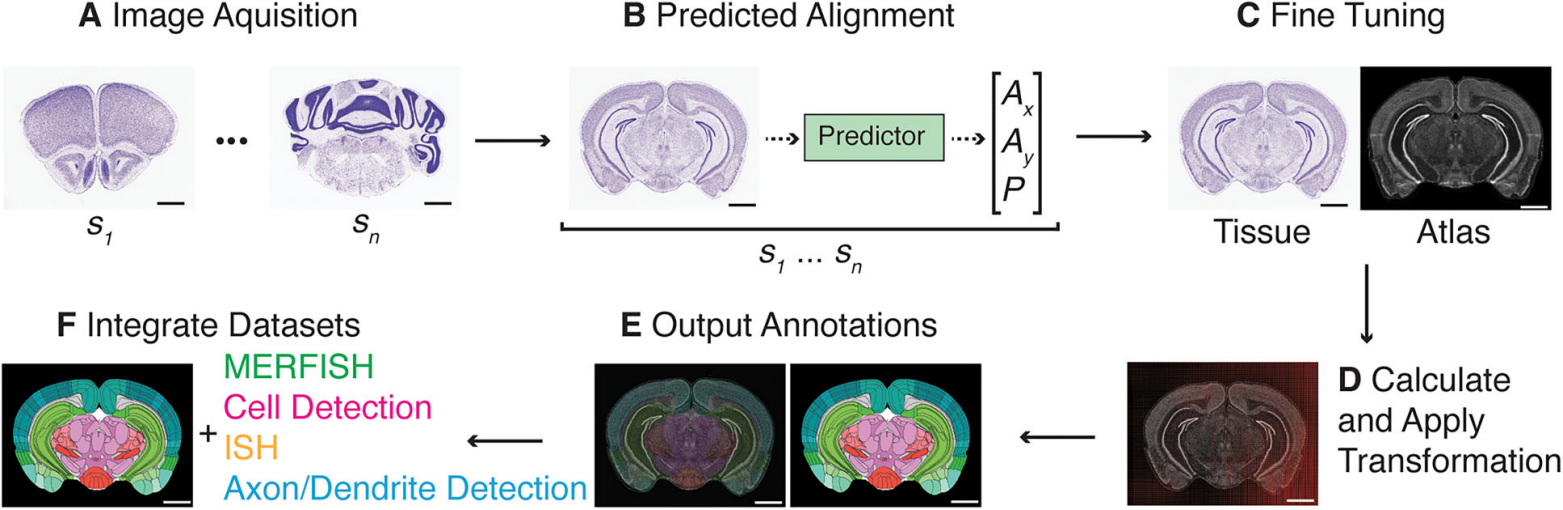
Bell Jar is a pipeline for aligning histology and integrating datasets. ***A***, Images of the counterstained mouse coronal brain sections with Nissl are acquired in sequence anterior to posterior. ***B***, The predictor network processes each image to produce a preliminary *x* cut angle, *y* cut angle, and anterior-to-posterior position (within the reference atlas). ***C***, Each prediction is evaluated for accuracy by a user and tuned for the best possible match. ***D***, An affine transformation followed by a B-spline transformation registering the atlas match onto the experimental tissue is found via gradient descent using SimpleITK (Lowekamp et al., 2013; Avants et al., 2014). ***E***, The transformation is applied to the annotations for the atlas match, resulting in the output annotation labeling the experimental tissue. ***F***, Annotations can be used to integrate a variety of datasets with the experimental tissue. Experimental tissue and atlas images were adapted from the Allen Mouse Brain Reference Atlas (Wang et al., 2020). Scale bars are 1 mm.

We first dissected the general workflow into its constituent steps to automate the analysis workflow. There are four key steps in going from raw images to results for a given experiment: prediction, alignment, detection, and integration (Fig. 1*B–F*). Each step outputs an analyzed component of the whole experiment that can provide a final result when combined with the others. Prediction determines the approximate location of a given section in the Allen Brain CCF. Alignment uses predictions to warp matching atlas borders onto the experimental tissue. Detection locates experimentally relevant signals in experimental tissue. Finally, integration combines the outputs of alignment and detection into usable experimental results (e.g., cell counts in a given region). An experiment can have any number of variations in its setup, so we begin our workflow assuming that the end-user has acquired serial sections of their tissue (Fig. 1*A*) and imaged them with a background stain and some signal of interest that can be separated into two distinct channels. Importantly, these must be serial coronal sections as they are currently the only supported data format, although they need not be whole brain sections, as single hemispheres, single pieces of cortex, and midbrain are supported. However, we plan to implement more experimental modalities like sagittal sections in future versions (see Discussion).

### The alignment pipeline

Alignment of experimental tissue sections begins with predictor network processing. The Allen Brain CCF (Wang et al., 2020) is a 3D volume, and our experimental tissue must have a corresponding section for alignment somewhere within that space. Therefore, we must define a good guess for the atlas section that matches each experimental tissue. The predictor network selects images to estimate each section’s *x* and *y* cut angles and the anterior-posterior positions (or *z*; Fig. 1*B*). The *x* and *y* angles are the rotation angles in the *x–z* and *y–z* planes, respectively, and the anterior-posterior position *z* is the depth in the coronal axis of the 3D volume. We include this step since typical microtome or cryostat tissue collection will result in some variation in cut angle. These values approximate how the section was cut during harvesting. The predictor network is based on the ResNet-101 architecture (He et al., 2016). The predictor was trained on a dataset of 1 million images generated from the Allen Brain CCF (Wang et al., 2020) 10 μm Nissl staining volume by slicing at random angles and depths coronally (single or dual hemisphere at random, 50:50) to produce a generalizable network that gives strong initial predictions. It uses a 256 × 256 coronal section image as input and outputs three normalized scalars representing the *x* and *y* cut angles and the anterior-posterior position. We repeat this process for all images in the experiment, then calculate the average predicted *x* and *y* cut angles and apply them across all predicted sections. Since these initial predictions are not perfect, we present the predictions to the user and enable tweaking on a section-by-section basis (Fig. 1*C*). We refer to this step as fine-tuning, and it is essential to incorporate human expert knowledge into the alignment process. The user examines each predicted section’s cut angle and position, ensuring they represent the experimental tissue reasonably and accurately. Once all sections have been checked, the workflow proceeds to the alignment step.

Registration of the experimental tissue to the Allen Brain CCF (Wang et al., 2020; alignment) is nontrivial. Experimental tissue can have widely ranging imaging settings and collection techniques, leading to significant inter- and intra-experimental differences in brightness, contrast, resolution, evenness of illumination, artifacts, and tissue quality. Previous methods avoid these variations using standardized imaging techniques like two-photon microscopy (Niedworok et al., 2016; Tyson et al., 2022) or deep learning approaches (Carey et al., 2023; Sadeghi et al., 2023) to make a generalized system insensitive to such factors. However, we used an image registration approach to achieve superior alignments at a local scale (cellular resolution) with standard 2D imaging. The moving image is the digital average tissue from the Allen Brain CCF, and the fixed image is our experimental tissue of interest. By identifying this transform, we simultaneously align the digital average CCF tissue with our experimental tissue and apply a corresponding transform to the CCF labels, as any changes applied to the digital tissue also applies to its labels. Transformations are found using a two-step composite registration process using the Python library for SimpleITK (Lowekamp et al., 2013; Avants et al., 2014). It relies on the inherent similarity between the gradient of the digital average Nissl image and the real tissue. Even though a user may not have background data that is Nissl stained, the digital average still represents a strong feature set to which we can compare against the experimental tissue. First, images are preprocessed by being resized to 360 × 360 pixels and converted from an intensity image into a gradient image via the Sobel operator (Van Der Walt et al., 2014; Virtanen et al., 2020). The gradient image is then normalized to ensure gradients between the experimental tissue and the digital average image are on the same scale. An initial affine transformation is found via gradient descent using Mattes mutual information (Mattes et al., 2001; Lowekamp et al., 2013) as the fit metric. After the affine transformation is found, the moving image is resampled by the affine transform, which centers it relative to the fixed image. A B-spline transformation is then found via gradient descent with the same metric, but this time, the resampled moving image is used to superimpose the digital average tissue on the fixed image (experimental tissue) accurately by matching gradient intensities (Fig. 1*D*). The same transformation of the digital average tissue is applied to its labels to create annotations for the experimental tissue (Fig. 1*E*). As a pixel-to-region mapping, such annotations could be applied to a variety of datasets, including fluorescently labeled signals to detect cells or molecules such as ISH and multiplexed error-robust fluorescence in situ hybridization (MERFISH) or any dataset in the tissue section’s coordinate space (Fig. 1*F*).

### Testing alignment outcomes

**Figure 2.**
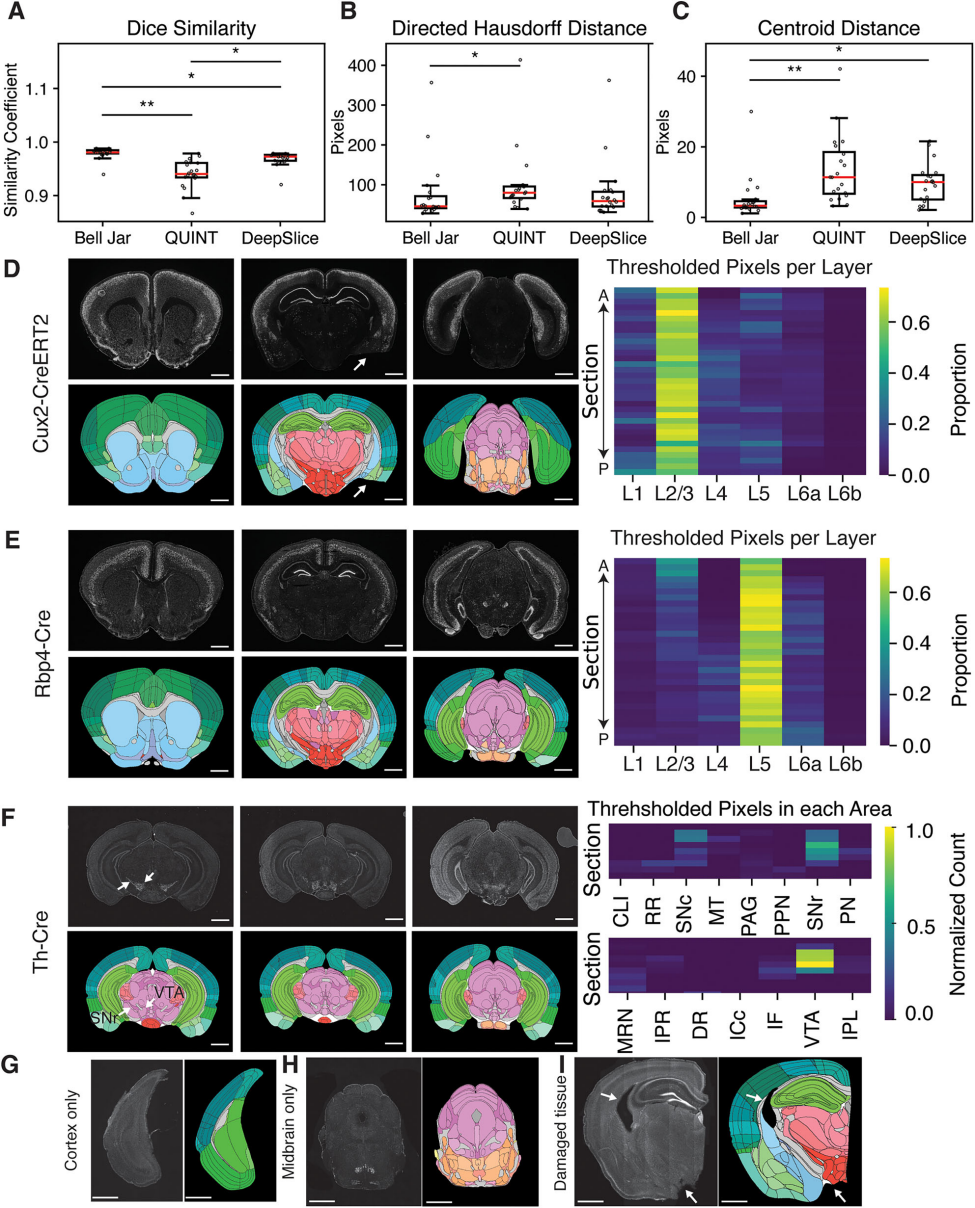
Bell Jar accurately assigns anatomical labels to experimental tissues. ***A***, The Dice similarity coefficient calculated between experimental tissue sections and their alignments produced from each method. Bell Jar outperformed QUINT (Yates et al., 2019) and DeepSlice (Carey et al., 2023; *p* = 4.65 × 10^−9^, 3.36 × 10^−3^, Kruskal–Wallis with post hoc Dunn, *n* = 20). ***B***, Directed Hausdorff distance between the contours of alignments and the aligned experimental tissue sections from each method. Bell Jar outperformed QUINT (*p* = 0.037, Kruskal–Wallis with post hoc Dunn, *n* = 20). ***C***, The distance between the centroids of experimental tissue sections and the alignments from each method. Bell Jar outperformed QUINT (Yates et al., 2019) and DeepSlice (Carey et al., 2023; *p* = 1.49 × 10^−4^, 6.32 × 10^−3^, Kruskal–Wallis with post hoc Dunn, *n* = 20). ***D***, Top, A selection of Cre ISH images from a Cux2-CreERT2 mouse provided by Allen Brain transgenic mouse characterization dataset (Lein et al., 2007). Allen Mouse Brain Reference Atlas, connectivity.brain-map.org/transgenic/experiment/571261835 (bottom) examples of per-section alignment alignments by Bell Jar. A heatmap shows the proportion of thresholded pixels (detected Cux2-Cre ISH signals) in each layer by section. The arrows in the middle panels point the damaged section images. ***E***, Top, A selection of Cre ISH images from an alignment of an Rbp4-Cre mouse provided by Allen Brain transgenic mouse characterization dataset (Lein et al., 2007). Allen Mouse Brain Reference Atlas, connectivity.brain-map.org/transgenic/experiment/117285137 (bottom) examples of per-section alignment alignments by Bell Jar. A heatmap shows the proportion of thresholded pixels (detected Rbp4-Cre ISH signals) in each layer by section. ***F***, Top, A selection of Cre ISH images from a Th-Cre mouse provided by Allen Brain transgenic mouse characterization dataset (Lein et al., 2007). Allen Mouse Brain Reference Atlas, connectivity.brain-map.org/transgenic/experiment/304164559 (bottom) examples of per-section alignment alignments by Bell Jar. Arrows show two key regions detected in our analysis VTA (ventral tegmental area) and SNr (substantia nigra, reticular part). A heatmap shows the normalized count of thresholded pixels (detected Th-Cre ISH signals) we detected in midbrain regions consistent with the expected expression. ***G***, An example of a single piece of cortex without midbrain or hindbrain aligned with Bell Jar. ***H***, An example of a single piece of midbrain without cortex aligned with Bell Jar. ***I***, Examples of damaged experimental tissue aligned by Bell Jar using masking. Arrows in ***D*** and ***I*** show damaged regions that were compensated for by masking. Scale bars are 1 mm. *p < 0.05; **p < 0.001; Kruskal–Wallis with post hoc Dunn.

We compared the efficacy of Bell Jar’s alignment against two contemporary tools: QUINT (Yates et al., 2019) and DeepSlice (Carey et al., 2023). To measure accuracy, we used the Dice similarity coefficient, which quantifies the overlap between the aligned sections and their corresponding atlas annotations. We found that Bell Jar’s performance in fitting anatomical labels to experimental tissue sections is superior, being consistently closer to the ground truth than that of QUINT or DeepSlice (Fig. 2*A*). Further comparison using the Directed Hausdorff distance, which assesses the maximum distance of a set to the nearest point in the other set, reinforced these findings. Bell Jar achieved a significantly lower mean distance than QUINT and was comparable with DeepSlice, indicative of more precise contour alignment (Fig. 2*B*). To further assess performance, centroid distance measurements between the experimental tissue sections and the respective alignments corroborated the superior performance of Bell Jar, as it yielded the smallest deviations (Fig. 2*C*).

To test Bell Jar’s alignment performance, we utilized ISH signals of Cre recombinase mRNAs in Cux2-CreERT2 mice expressing Cre in the cerebral cortical layers 2 and 3 mostly (Lein et al., 2007). We investigated whether Bell Jar can accurately detect Cre expression at the expected localization across different sections (Fig. 2*D*). We first aligned all the sections using Bell Jar and then used the resulting annotations to find the proportion of Cre ISH signals in the cortical layers of each section. A positive ISH signal was defined as pixels above an intensity threshold. As expected, the signal is predominantly in layers 2 and 3 (Fig. 2*D*). We also used different Cre ISH expression images of two transgenic mice, Rbp4-Cre and Th-Cre. Rbp4-Cre mice express Cre specifically in the cortical layer 5, and Th-Cre mice in midbrain structures such as the ventral tegmental area (VTA) and substantia nigra (SNr; Lein et al., 2007; Fig. 2*E*,*F*). Bell Jar successfully aligns the experiment brain tissue images from these two transgenic lines based on the precise demarcation of Cre+ cortical layers and midbrain subregions (Fig. 2*E*,*F*).

We also investigated whether Bell Jar could handle challenging tissue types that can be produced when the posterior portions of the brain are coronally sectioned. Bell Jar can align a single piece of cortex or midbrain section without midbrain (Fig. 2*G*) or other forebrain tissues (Fig. 2*H*), respectively. It also showed versatility in aligning damaged tissues by employing masking techniques, removing portions of the atlas image to better match the experimental one, and ignoring areas of experimental artifact or damage (Fig. 2*I*). These tests underscore the software’s adaptability to various experimental conditions and its proficiency in maintaining alignment accuracy even in nonideal samples.

### Benchmarking integrated cell detection

**Figure 3.**
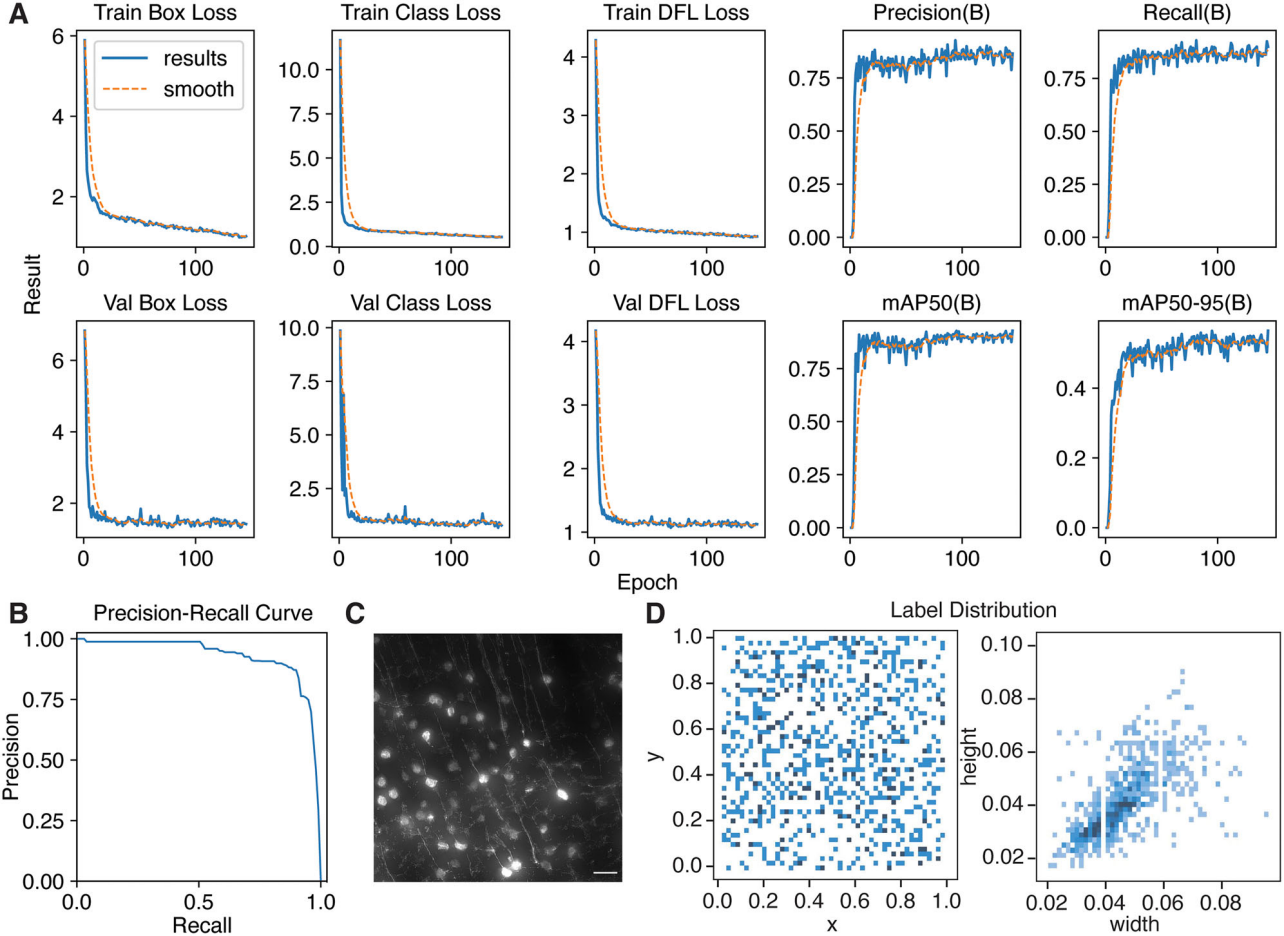
Bell Jar uses object detection to detect cells automatically. ***A***, Training results for the YoloV8 (Jocher et al., 2023) object detector Bell Jar uses for cell detection. Each graph represents a measurement of model training progress. The final model demonstrates minimal loss and high precision. ***B***, The precision-recall curve for the cell detection model. ***C***, A representative microscopic image of the training data was used to create the cell detection model, showing RV*dG*-mCherry labeled neurons in the mouse visual cortex. The scale bar is 50 μm. ***D***, Heatmaps depicting the distribution of annotated cells from the training dataset in normalized *x* and *y* coordinates (left) and in normalized cell body widths and heights (right).

Bell Jar provides integrated cell detection by object detection for identifying cells in the experimental tissue. We leverage the YoloV8 (Jocher et al., 2023) object detection model to autonomously detect cell bodies, demonstrating a notable advantage in handling varying signal levels, a challenge that traditional thresholding methods often fail to address (Fig. 3*A*). The precision-recall curve for the cell detection model indicates high reliability, showcasing the model’s ability to effectively discern true positives from false positives (Fig. 3*B*). The model was trained on a representative dataset of RV*dG* (G-deleted rabies virus) traced neuronal cell bodies (Fig. 3*C*). Our training dataset is composed of 700 annotated cells with different *x* and *y* positions in 41 curated images (Fig. 3*D*, left). These annotated neurons also exhibit a broad range of cell body widths and heights (Fig. 3*D*, right), all of which helps generalize the model. From our initial 700 cells, we further augmented the dataset with random brightness and saturation levels to simulate a variety of imaging parameters (the final training had 2,600 labeled instances). In addition to these advantages, Bell Jar also uses slicing-aided hyper inferencing (SAHI; Akyon et al., 2021) to enhance its detections. It tiles high-resolution input images into small overlapping tiles (640 × 640, 50% overlap) and performs detection on each of them and then aggregates results by non-maximum suppression of overlapping detections. Using SAHI ensures that cells are detected at a contextually relevant scale since each cell body may be as little as 200 px in a high-resolution microscopy image.

**Figure 4.**
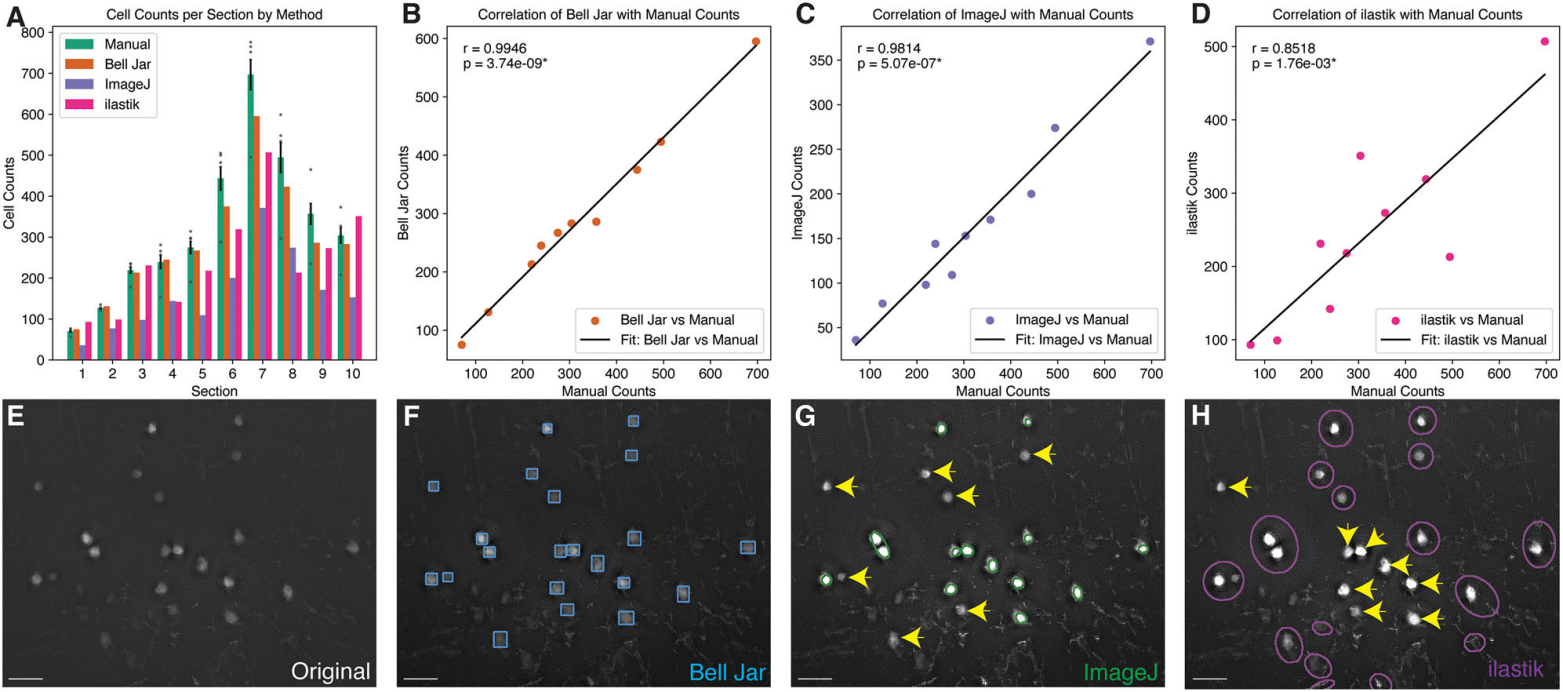
Bell Jar cell counting is comparable with manual counting by an experienced rater and outperforms standard methods. ***A***, Counts of neurons detected in ten representative coronal sections of a mouse with cortical cells traced by RV*dG* by each tested method. ***B***, Correlation of Bell Jar automatic counts and manual counting across 10 representative sections (*r* = 0.9946, *p* = 3.74 × 10^−9^, Pearson’s correlation). ***C***, Correlation of ImageJ (Schindelin et al., 2012) automatic counts by thresholding and manual counting across ten representative sections (*r* = 0.9814, *p* = 5.07 × 10^−7^, Pearson’s correlation). ***D***, Correlation of ilastik (Berg et al., 2019) automatic counts and manual counting across 10 representative sections (*r* = 0.8518, *p* = 1.76 × 10^−3^, Pearson’s correlation). ***E***, The original sample image of labeled mouse cortical neurons. ***F***, Bell Jar’s detections of neurons are represented on the sample image as blue bounding boxes. ***G***, ImageJ’s detections of neurons are represented on the sample image as green circles. ***H***, ilastik’s detections of neurons are represented on the sample image as purple circles. Yellow arrows indicate missing detections of cells in ImageJ and ilastik (***G***, ***H***). The scale bars are 50 μm.

Bell Jar’s cell counting performance was benchmarked against manual counts and standard automated methods, such as ImageJ (Schindelin et al., 2012) and ilastik (Berg et al., 2019). We selected tissue sections from experiments previously imaged in our lab with rabies virus-traced neurons in the mouse cerebral cortex labeled with mCherry. All the sections were counted with each method independently, and the counts were compared with each other (Fig. 4*A*). The results demonstrated that Bell Jar’s deep learning object detection-based cell counting closely aligned with manual counts across 10 representative sections, evidenced by significant correlation (ImageJ vs Manual: *r* = 0.9814, *p* = 5.07 × 10^−7^; ilastik vs Manual: *r* = 0.8518, *p* = 1.76 × 10^−3^; Bell Jar vs Manual: *r* = 0.9946, *p* = 3.74 × 10^−9^; Fig. 4*B*). As performed by Bell Jar, the neuron counts per section closely matched those of manual counting, outperforming the counts achieved by ImageJ and ilastik, which rely on intensity thresholding and machine learning approaches, respectively (Fig. 4*A*,*B*). In contrast, counts obtained from ImageJ and ilastik, while still correlated with manual counts, showed lower correlation coefficients, suggesting less accuracy with the manual standard (Fig. 4*C*,*D*), mainly because ImageJ and ilastik mislabeled neurons (Fig. 4*G*,*H*, yellow arrowheads). Direct comparison with these conventional methods suggests the robustness of Bell Jar’s automated cell counting method.

## Discussion

### Bell jar advances histological analysis through automation

The integration of Bell Jar into histological analysis represents a significant advancement in the automation of tissue alignment by using automated nonlinear deformations for image registration. The alignment module’s ability to accurately map experimental tissue to a reference atlas not only streamlines the initial stages of analysis but also ensures reproducibility. This yields a particularly notable advantage when considering the variance in signal levels and tissue integrity commonly encountered in experimental settings. Bell Jar’s alignment outcomes, validated against other current state-of-the-art methods such as DeepSlice and QUINT (Yates et al., 2019; Carey et al., 2023), demonstrate its robustness and reliability. Together, these improvements enhance confidence in subsequent analytical processes.

### Bell Jar enhances precision in cell detection

Applying the YoloV8 (Jocher et al., 2023) object detection algorithm within Bell Jar for cell counting is a methodological shift from traditional thresholding-based techniques to a more sophisticated, deep learning-based approach. This shift is substantiated by the high correlation of Bell Jar’s cell counts with manual cell counts. These findings suggest that the object detection method employed by Bell Jar is sensitive enough to replicate the discernment of trained human eyes. Furthermore, the precision-recall balance achieved by the model underscores its efficiency in distinguishing true cell bodies from noise, a task that has been challenging for automated systems.

### Implications for neuroscience and beyond

The implications of Bell Jar can extend into various fields of other biological research, where histological analysis is a cornerstone in other tissue systems or model organisms. The high-throughput and automated nature of Bell Jar’s pipeline enables researchers to process large datasets with greater ease, facilitating more extensive studies that the laborious nature of manual analysis may have previously limited. In neuroscience, this could accelerate the precise mapping of neural circuits and quantifying neuron populations across different brain regions.

### Limitations of the current work

Despite the strengths of the Bell Jar system, certain limitations must be acknowledged. Bell Jar requires comprehensive section images in both the counterstain, such as Nissl or DAPI, and signal channels simultaneously, an imaging paradigm that may not align with the objectives or resources of every study. Researchers who only image specific regions of interest, such as a subregion of the hippocampal formation, may find this requirement burdensome, as it necessitates comprehensive imaging beyond their targeted area. Moreover, obtaining images of the sample with consistent aspect ratios in both channels is critical for ensuring accurate alignment. This can pose a practical challenge, mainly when dealing with samples that are difficult to image in their entirety or when equipment limitations impact image consistency.

Additionally, while Bell Jar substantially reduces the need for expert input compared with traditional manual methods, it does not eliminate it. The initial setup and some aspects of the alignment process still require user intervention and a degree of expertise, while this user intervention provides the opportunity to correct errors and improve the qualities of the image alignment. This necessity for expert input, although reduced, could introduce user bias and inconsistency, especially in cases where multiple individuals are involved in the image processing workflow.

Lastly, while object-based cell detection offers a clear advantage over thresholding-based methods, it does require some parameter tuning to prevent excessive false positives. Lowering the confidence threshold may detect more cells but at the risk of false-positive detections. A key challenge is balancing confidence cutoffs for predictions to limit these false positives and having a sensible threshold to label most cells in an image. However, the end user can resolve these values on a per-experiment basis.

### Future directions

The Bell Jar platform offers a foundation for further methodological enhancements and broader applications. The adaptability of the software to various tissue types and staining methods suggests its potential utility in a broader range of biological studies. The underlying algorithms could also be refined to include more advanced artificial intelligence techniques, improving accuracy and expanding the scope of detectable features within tissue sections. One of the most likely future uses for Bell Jar is its potential integration into spatial transcriptomic studies, where histological data can be combined with genomic data. This comprehensive approach could provide greater accuracy in the anatomical localization of transcriptomic data.

In conclusion, Bell Jar represents a step forward in automated histological analysis. Its ability to provide accurate, reproducible results lays a convenient platform for neuroscience research characterized by high-throughput image data analysis. Likewise, Bell Jar’s open-source nature lends itself to cross-disciplinary integration. Bell Jar provides easy access for neuroscientists to analyze the mouse brain anatomy accurately and flexibly. Additionally, this method can be applied to any digital 3D reference atlas. So, in the future, support may be added for other model organisms (e.g., rats) which have defined atlases.

## Data Availability

This study did not generate new unique biological reagents. Microscopy and any additional data reported in this paper will be shared by the lead contact upon request. The code/software described in the paper is freely available online at https://github.com/asoronow/belljar/releases/tag/v10.1.0

10.1523/ENEURO.0036-23.2025.d1 Extended Data 1 The step-by-step guide for Bell Jar. Download Extended Data 1, DOCX file.

## Synthesis

Reviewing Editor: Silvia Pagliardini, University of Alberta Decisions are customarily a result of the Reviewing Editor and the peer reviewers coming together and discussing their recommendations until a consensus is reached. When revisions are invited, a fact-based synthesis statement explaining their decision and outlining what is needed to prepare a revision will be listed below. The following reviewer(s) agreed to reveal their identity: Yongsoo Kim.

Authors did a great job to improve the software and the manuscript. My data scientist downloaded the package and confirmed that it works as claimed. Moreover, the user tutorial is comprehensive and user-friendly with example datasets. This mitigated the prior concern about the usability of the software. Comparison to existing software with quantitative assessment is great. This software can help biologists without expert coding ability to perform image analysis with microscopic slides with the CCF alignment, which will be highly useful. Overall, the manuscript is well-written while clearly acknowledging the current limitations and future direction. I do have one comment to further improve the manuscript.

Fig3D: I have a hard time understanding this figure. Is this detection result from Fig3C, or something else? The related text in the result section was not clearly written to specify what this data represent.

## Author Response

Response to the reviewers (eN-OTM-0036-23R1) We thank the reviewers and the editor for their positive evaluation of our manuscript. We respond to specific comments in the context of the reviews below using a blue font color.

Synthesis of Reviews:

Significance Statement Comments for Author (Required): n/a Comments on the Visual Abstract for Author (Required): n/a Synthesis Statement for Author (Required):

Authors did a great job to improve the software and the manuscript. My data scientist downloaded the package and confirmed that it works as claimed. Moreover, the user tutorial is comprehensive and user-friendly with example datasets. This mitigated the prior concern about the usability of the software. Comparison to existing software with quantitative assessment is great. This software can help biologists without expert coding ability to perform image analysis with microscopic slides with the CCF alignment, which will be highly useful. Overall, the manuscript is well-written while clearly acknowledging the current limitations and future direction. I do have one comment to further improve the manuscript.

Fig3D: I have a hard time understanding this figure. Is this detection result from Fig3C, or something else? The related text in the result section was not clearly written to specify what this data represent.

Thank you for pointing out this. We would like to clarify that Figure 3D does not depict the detection results from Figure 3C. Instead, Figure 3D illustrates the characteristics of annotated cells in our training dataset, specifically their x and y positions within the images (Figure 3D, left) and their widths and heights (Figure 3D, right).

To address this, we have revised the text as follows:

In the Results section (line 366), we have updated the sentence to: "Our training dataset is comprised of 700 annotated cells with different x and y positions in 41 curated images (Figure 3D, left). These annotated neurons also exhibit a broad range of cell body widths and heights (Figure 3D, right), all of which helps generalize the model." In the Figure Legend (line 606), we have modified the description to: "(D) Heatmaps depicting the distribution of annotated cells from the training data set in normalized x and y coordinates (left) and in normalized cell body widths and heights (right)."
