# Supplementary material for: Bell Jar: A Semiautomated Registration and Cell Counting Tool for Mouse Neurohistology Analysis
Source: eNeuro. 2025 Feb 3;12(2):ENEURO.0036-23.2025. doi: 10.1523/ENEURO.0036-23.2025 (PMC11801229; doi:10.1523/ENEURO.0036-23.2025)
Supplement: Extended Data 1 — The step-by-step guide for Bell Jar. Download Extended Data 1, DOCX file. [file eneuro-12-ENEURO.0036-23.2025-s002.docx]

Bell Jar Guide (version v10.1.0)

**0. Installation** https://github.com/asoronow/belljar/releases

Download the package for your operating system, decompress it, and run the belljar executable/app. You must ensure the folder that the executable/app is located in has no special characters or spaces in its path.

For Mac, download x64 dmg.

For Windows download, win32-x64.

*Installation of Bell Jar will take approximately 30 minutes, assuming at least 10 MB/s download speeds, and requires ~5 GB of storage.*

Successful Installation will show an entry screen with several options: select “Menu” to get started or “Open Guide” to view this guide from Bell Jar.


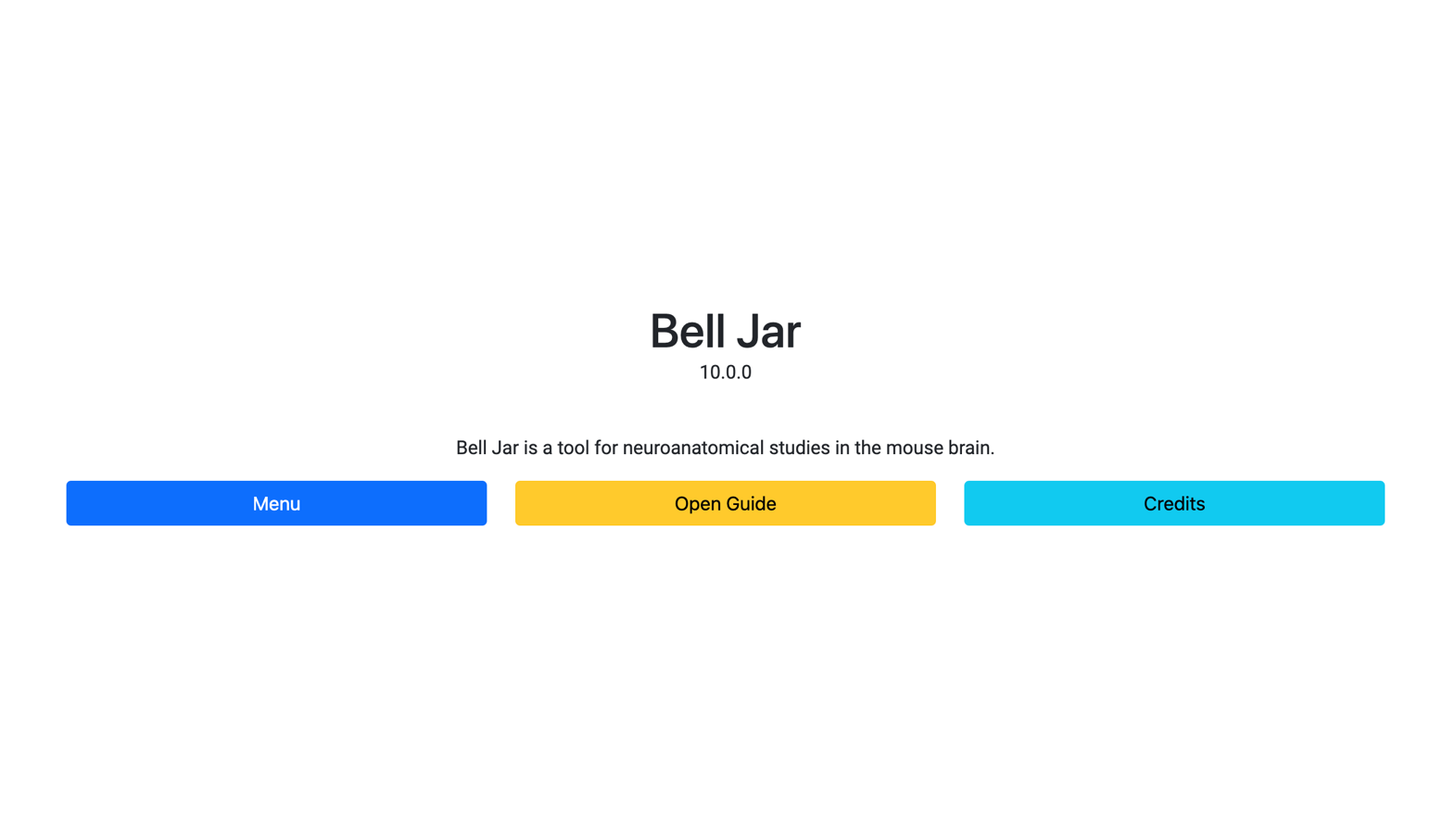


Once “Menu” is selected, three sets of tools (1. Preprocessing, 2. Prediction, 3. Analysis) are displayed.


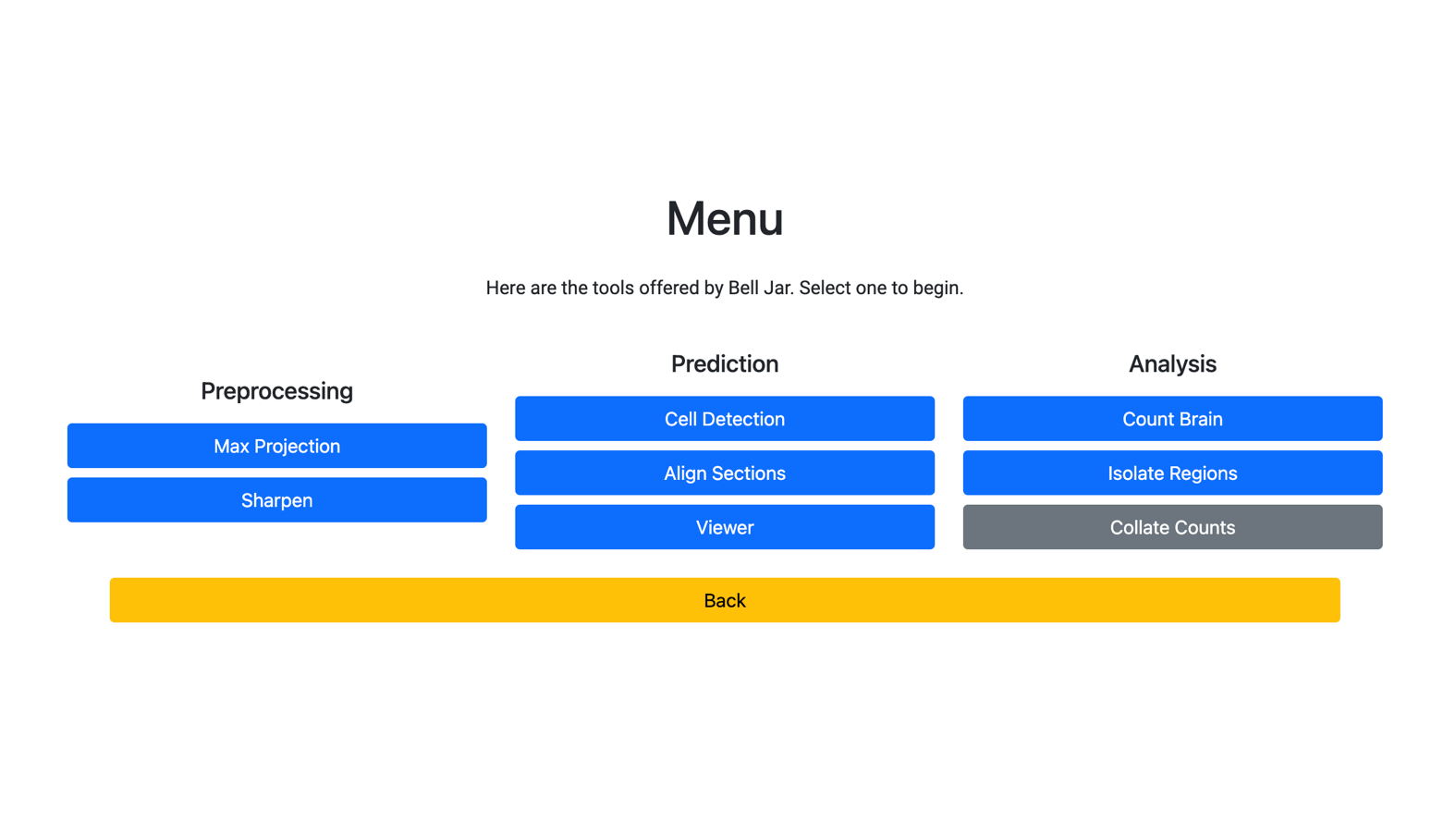


We recommend the users go to section 4, “Processing the sample dataset,” for a quick example run-through on a sample dataset before running the tools described below.

# 1. Preprocessing

The preprocessing tools (1a-Max Projection and 1b-Sharpen) are helpful runtimes to prepare images for the detection steps such as 2a-cell detection.

## 1a. Max Projection

It takes a directory of z-stacked single-channel images and flattens it into a single plane by taking only the pixels of the greatest intensity. This function offers a fast way of “focusing” images with focal depth.

Usage:

1. Create a new directory for the output of the process. (e.g., Animal42_maxprojections)
2. Select the input path to your z-stacked images. They must be in the tiff (ometiff works as well) file format.
3. Select the output path as the directory you created.
4. Click ‘Run’ to initiate the progress and monitor results by the loading bar on the bottom of main window or the output folder contents.

Outputs: Max projected tiff files in your output path directory.

## 1b. Sharpen

It takes a directory of flat images, applies an unsharp mask of the selected kernel parameters to each image, and (optionally) performs adaptive histogram equalization. This process helps remove blur and illumination artifacts during EFI (Extended Focus Imaging) processing and max projection.

Usage:

1. Create a new directory for the output of the process. (e.g., Animal42_Sharpened)
2. Select the input path to your flattened images. They must be in the tiff (ometiff works as well) file format.
3. Select the output path as the directory you created.
4. Input a radius size that will define the kernel size of the filter. A good starting point is 3px; higher values may begin to create undesirable artifacts but may be necessary on very blurry images.
5. Set an amount value. This weights how the sharpness kernel will be applied to your image the default value is 2.
6. Click ‘Run’ to initiate the program and monitor results by the loading bar at the bottom of the main window or the output folder contents.

Outputs: Sharpened tiff files in your output path directory.

# 2. Prediction

The prediction tools (2a. Cell Detection, 2b. Align, 2c. Viewer) comprise the main components of Bell Jar. They can align tissue section images to the Allen brain CCFv3 (http://atlas.brain-map.org/), detect labeled neurons in tissue sections, and integrate these two sets of data into detailed counts of cells by brain region in an experiment.

## 2a. Cell Detection

The ‘Cell Detection’ tool finds cells (cell bodies) in flattened (single plane) tissue sections. The detector was trained on the fluorescently labeled cell bodies traced by G-deleted engineered rabies tracer (see Method sections of Soronow et al., TBA). It can also be used on multi-channel images to get co-localization data in the detection step.

Usage:

1. Create a new directory for the outputs of the process (e.g., Animal42_detections).
2. (Optional) Prepare your images as multi-channel tiffs where each channel is a separate signal channel with cell bodies you wish to count and check ‘Multichannel’ in advanced settings.
3. Prepare your input images in anterior to posterior order along the coronal sections with ascending section indicator names (e.g., Animal42_section1, Animal42_section2); the ordering and naming ensure that the predictions are processed in the same order as the other analysis steps.
4. Select the input path to your preprocessed images with cells to detect.
5. Select the output path to the new directory you created.
6. (Optional) Under advanced settings, you can specify a custom YOLOv8 model. These advanced settings allow you to adjust the tile size and confidence threshold.
7. Click ‘Run’ to initiate the program and monitor the results by the loading bar or the output folder contents.

Outputs: Detection points maps (for use with traditional segmentation analysis tools), bounding box images (to verify detections), and raw predictions (saved as .pkl files, used in subsequent analysis steps) in your output path directory.

## 2b. Align

The ‘Align’ tool takes in a directory of DAPI or Nissl-stained section images and assists the user in aligning them to the Allen Brain atlas CCFv3.

**Necessary:** The input images here should correspond directly to the prediction images (e.g., Animal42_section1 should be the name of both the DAPI/Nissl section (background staining) and the image with cells present (the experimental image). They must be **.PNG** format 8 bit and **NO** larger than **2MB,** or alignments may never finish.


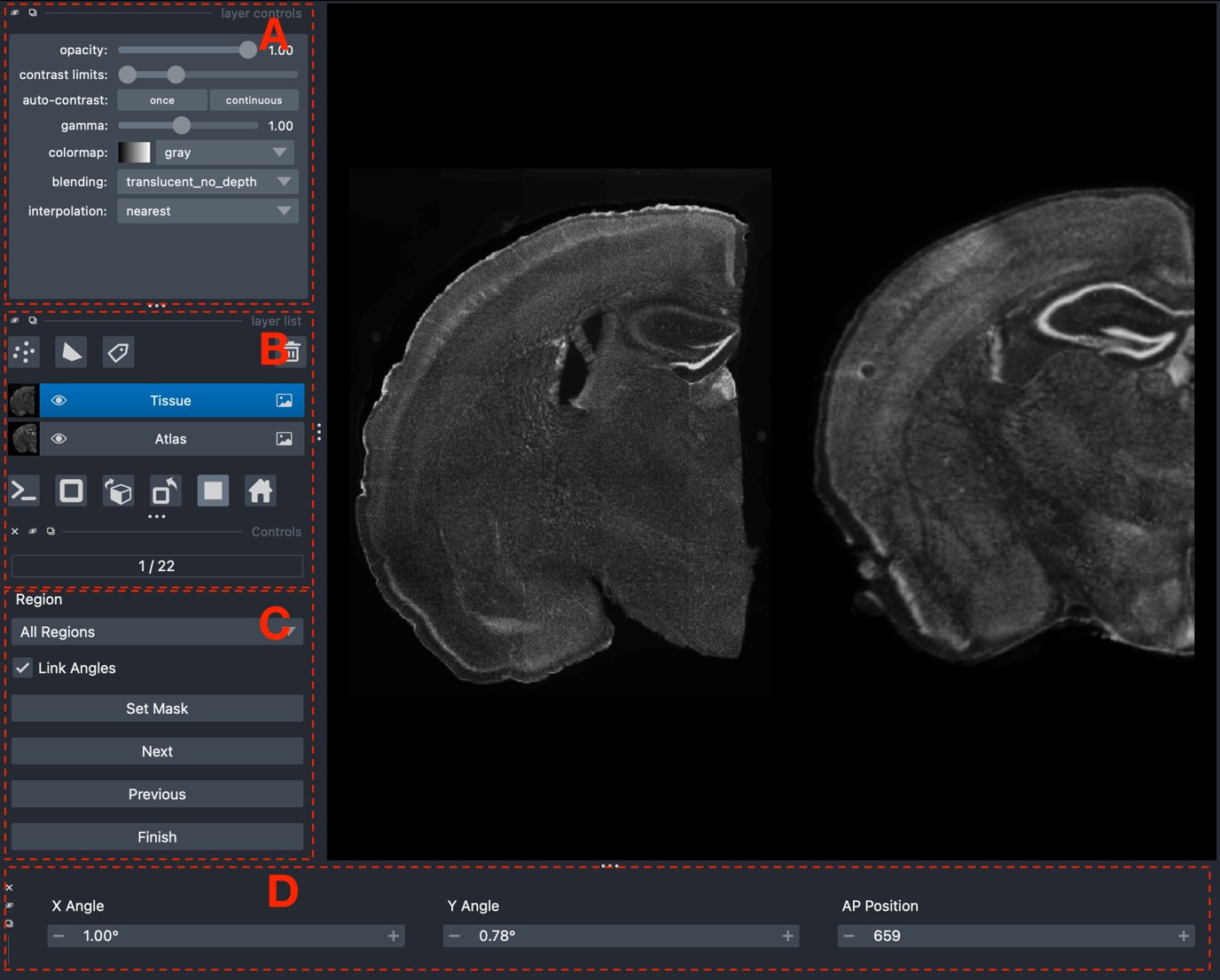

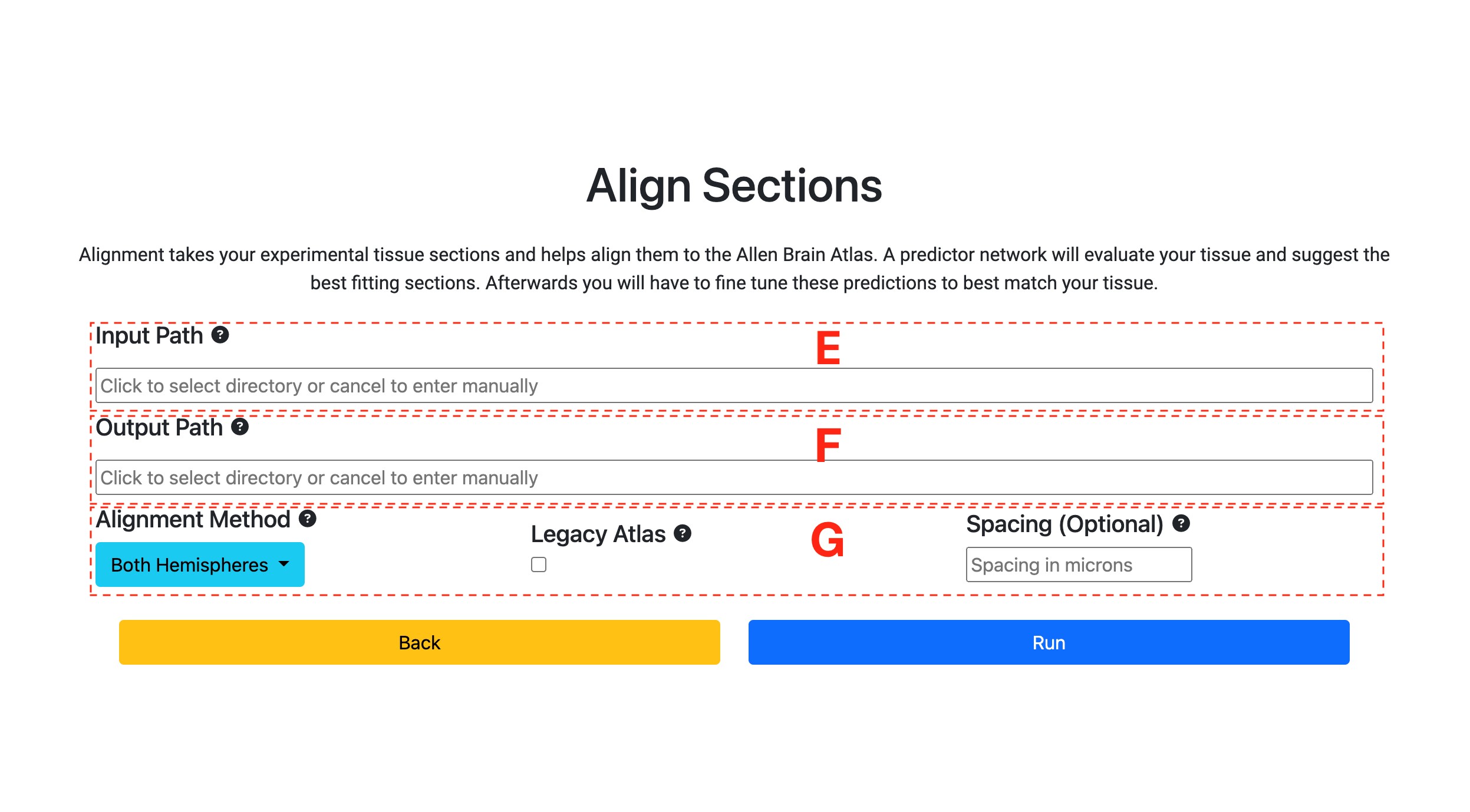


Usage:

1. Create a directory for the outputs of the process. (e.g., Animal42_alignment)
2. Prepare your section images in anterior to posterior order with ascending section indicator names (e.g., Animal42_s001, Animal42_s002); the ordering and naming ensure that the alignments are processed in the same order as the other analysis steps. (section images should be 8-bit PNGs)
3. Select the input directory to your section images (E)
4. Select the output path to the new directory you created (F). (e.g., Animal42_alignment)
5. Select whether the input images are brain sections containing ‘Both Hemispheres’ or a ‘Single Hemisphere’ (G) (if you select ‘Single Hemisphere’, your section images must be in a normal coronal left hemisphere orientation).
6. (Optional) You can input the spacing between your sections in microns (G) to assist the prediction process.
7. Click ‘Run’ to initiate the program.
8. During execution, the script will first predict your samples' cut angle and position. Afterward, a new window will pop up, prompting you to fine-tune (if you do not see this window, it may be hidden or minimized. Check the taskbar/dock).
9. (Optional) Set the ‘Atlas’ image in the bottom left layer selector (B) to a different color and adjust the opacity (top left under ‘layer controls,’ A) until you have reached visibility you are comfortable with and can easily be compared with your section images.
10. You must tune each atlas image to best match your section image by using the angle and position options (D) to move anterior to posterior through the reference atlas and adjust the cut angle. Cut angles are linked by default, changing all sections; if you want to adjust a single section, uncheck the ‘Link Angles’ box (C).
11. (Optional) If the cerebral cortex in any section is isolated from the midbrain or vice-versa, you can align them alone using the ‘Region’ selector (B) and selecting ‘Cerebrum Only’ or ‘No Cerebrum.’ Normal tissue with separation (e.g., a gap from mounting) should be addressed by masking ‘Set Mask,’ (B) which lets you erase portions of the atlas image to better match your tissue and improve alignment.
12. Once satisfied with your selected match, click ‘Next’ to move on to the next section.
13. Repeat this process until you arrive at your final section image, and then click ‘Finish’ to write the results (C).
14. Monitor the output folder to check the progress of the resulting output. The warping will take 5-15 minutes per section, depending on your computer’s speed (the average PC will take 30 minutes per section).

Outputs: Reference atlas slices, Composite maps for each section, and Raw annotation data (as .pkl files for subsequent analysis) in your output path directory (e.g., Animal42_alignment).

## 2c. Viewer

The ‘Viewer’ tool takes in a directory containing the annotations and DAPI/Nissl section images from alignment, allowing users to adjust the alignments. It can also be used to analyze signal images with overlaid maps as long as they have the same aspect ratio as the aligned images.

**Important:** This routine directly modifies the annotations. Please follow the setup instructions. Additionally, a mouse (not a trackpad) is **STONGLY** advised.


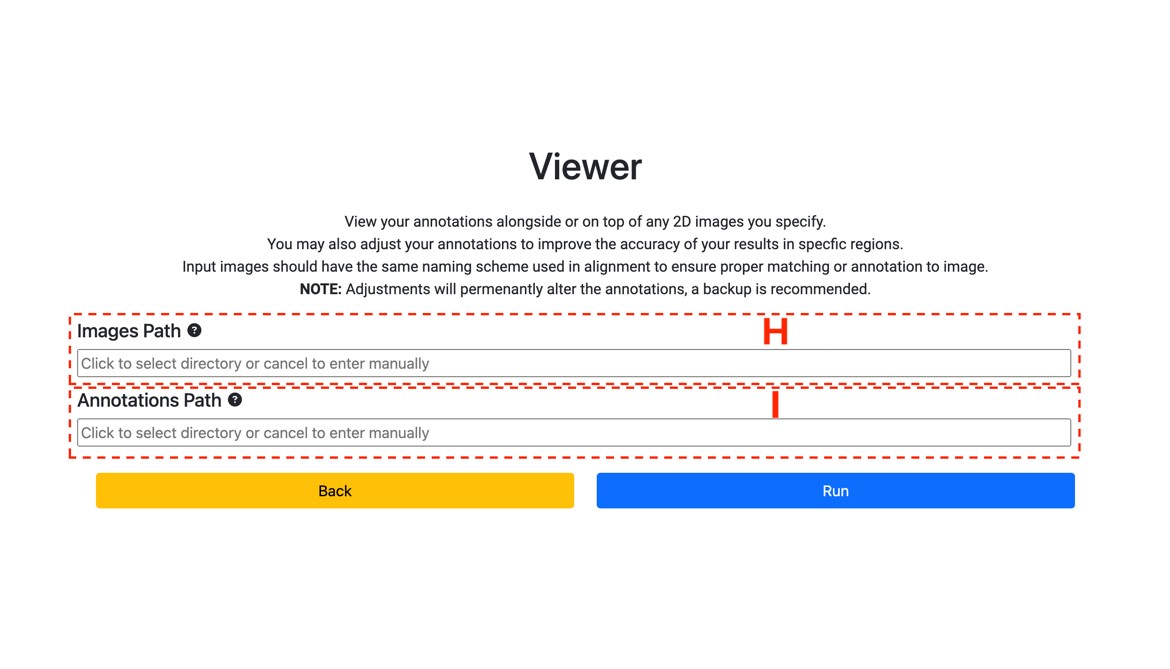


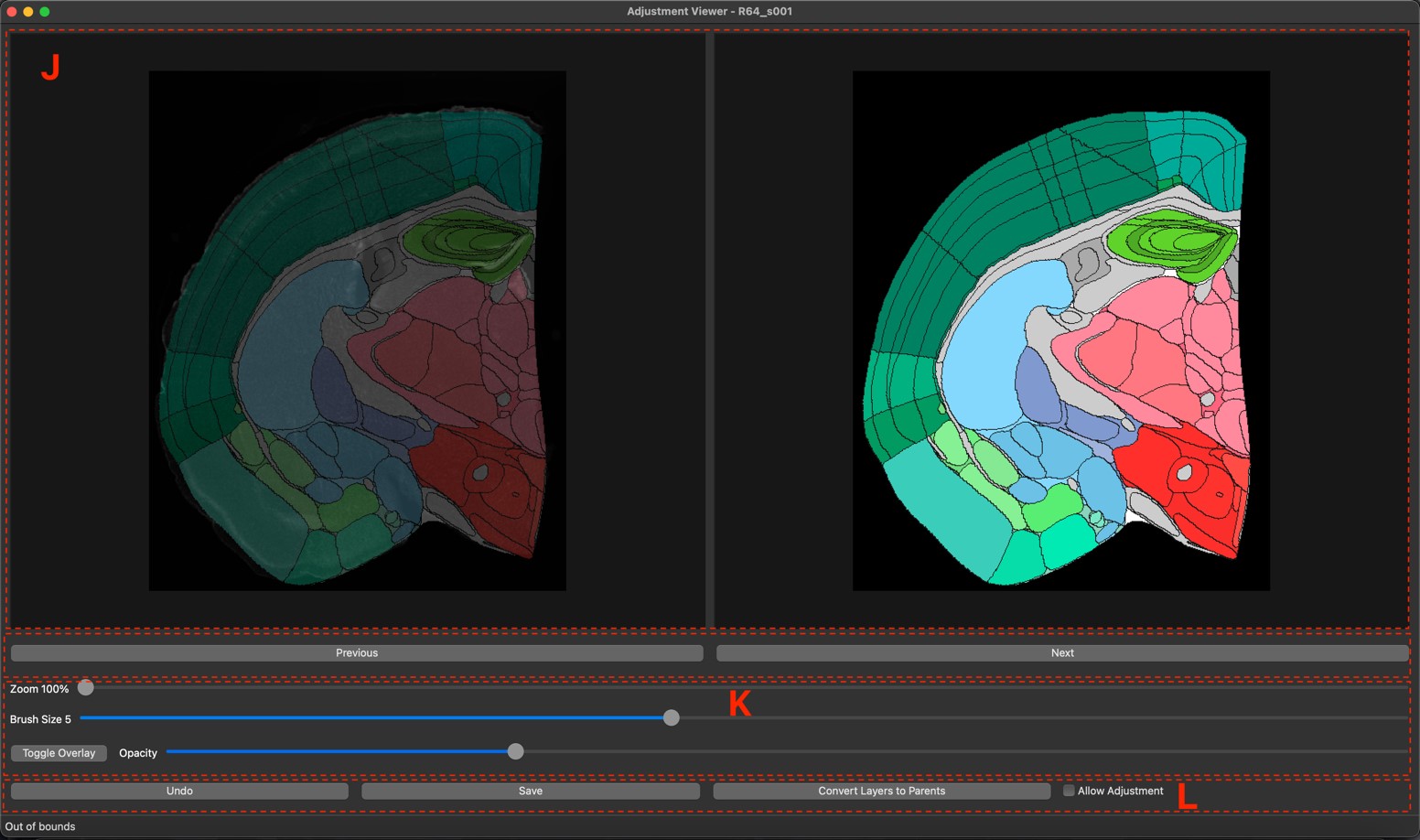


Usage:

1. Create a new directory clearly labeled as adjustments (e.g., Animal42_adjusted).
2. Make a copy of the annotations (.pkl files from the alignment step) in the new directory (preserve naming).
3. Select the input path to your images (stain or signal, H).
4. Select the new adjustment director you just created (I).
5. Run the program.
6. The viewer window will open with your section into the left (J) and the map to the right (J). An overlay can be toggled onto your section using the toggle overlay button (K).
7. You can modify the annotations by right-clicking any region on your section to select it. Your selected region and the region currently below your mouse cursor appear in the status bar below (L; in this image, it says ‘Out of Bounds’). Before any modifications can be painted on you must check the ‘Allow Adjustment’ checkbox (L).
8. You can undo any changes you make sequentially with the ‘Undo’ button (L) and save your changes with the ‘Save’ button (L).
9. When you finish a section, the ‘Next’ and ‘Previous’ buttons let you navigate your data.
10. You can close the window when you’re finished and have saved all changes.

Outputs: Modified annotation .pkl files in the selected input directory.

# 3. Analysis

The output tools (3a. Count brain, 3b. Isolate Regions, 3c. Collate Counts) produce useful output from the Bell Jar prediction routines. Currently, it consists of one primary tool ‘Count Brain’ we have developed that counts all the cells in an experiment processed with the prediction tools. Isolate regions and Collate Counts are experimental and legacy tools respectively and are not for general use.

## 3a. Count brain

Takes in the raw cell location predictions and raw annotations to produce a count of predicted labeled cells per region in the experimental brain. Counts are output by section and as totals in a CSV file.

Usage:

1. Create a new directory for the outputs of the process. (e.g., Animal42_count).
2. Select the directory where your raw prediction pkls are located as the predictions path (e.g., Animal42_detections).
3. Select the directory where your raw annotations pkls are located as the annotations path (if you adjusted your annotations, select the adjustments directory, e.g., Animal42_alignment or Animal42_adjusted).
4. Select the new directory you created as the output path.
5. Run the program and check the loading bar or monitor the output folder for the result.

# 4. Processing the sample dataset

We have provided a minimal example case of using Bell Jar to align the sections to the Allen brain CCFv3 and count the cells by region in some experimental tissues. Please first download the sample dataset from [here](https://storage.googleapis.com/belljar_updates/belljar-sample.zip) and unzip it. All directories have been created for you, and the sample images have been preprocessed. At each step, please return to the instructions in the guide for running it.

Raw sample link: https://storage.googleapis.com/belljar_updates/belljar-sample.zip

Click ‘Menu’ on the home screen.


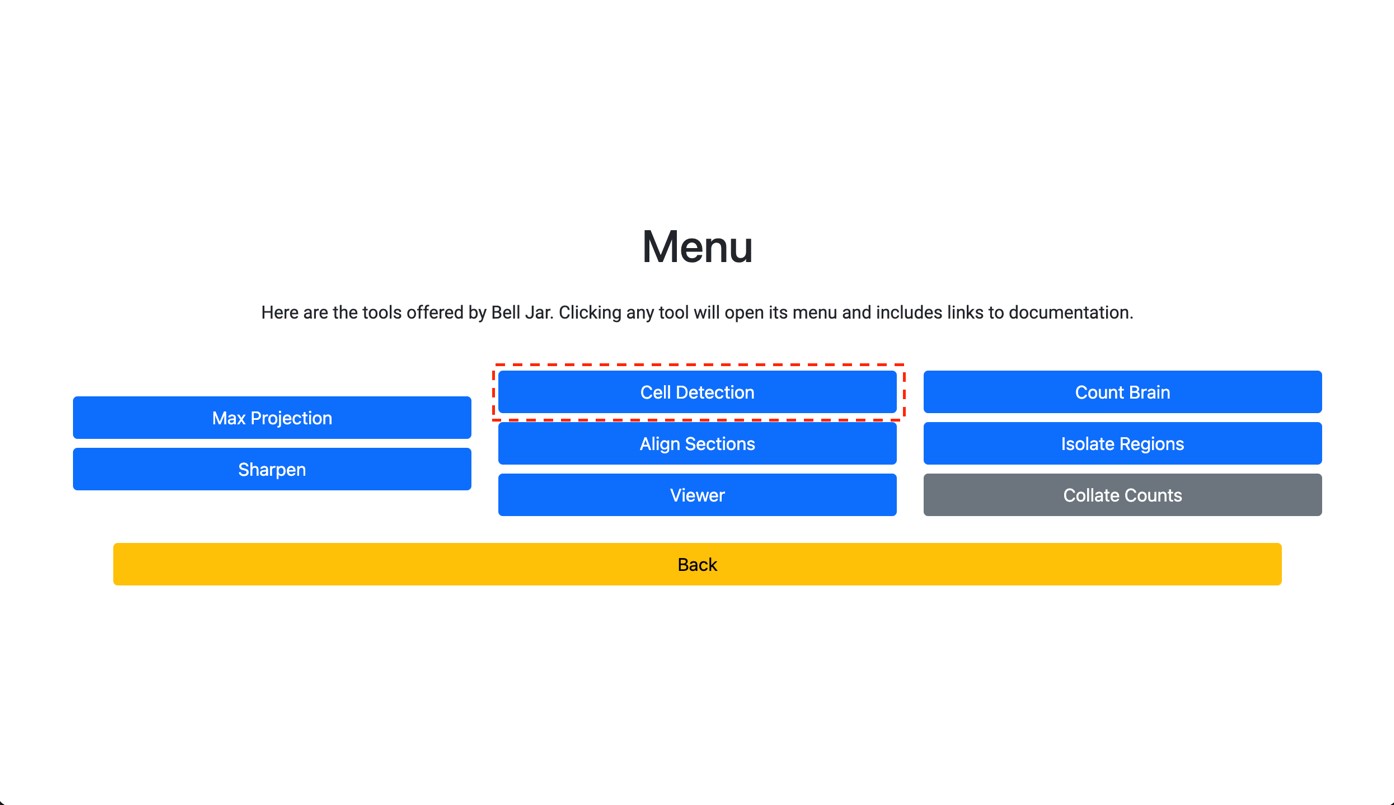


1. Begin by clicking to run ‘Cell Detection’ on the “Cell Images” in 'belljar-sample’ folder provided to obtain detections. Specify Input Path as a folder where the image files are located (e.g. ~/belljar-sample/Cell Images) and Output path as a folder to store the processed images (e.g. ~/belljar-sample/Detections).
2. If cell detection is completed, move to ‘Aligning’ step (#3).


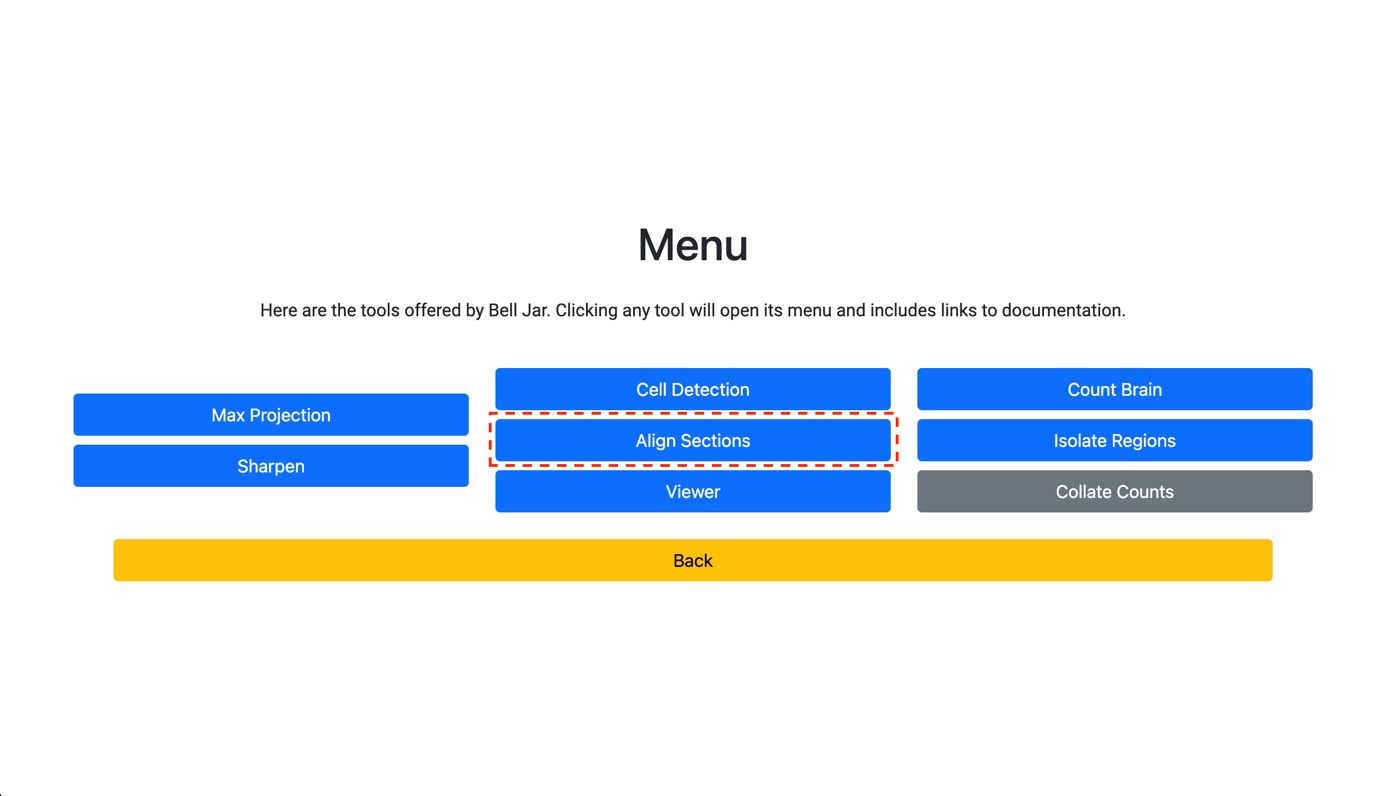


1. Run the aligning step on the provided “DAPI images” to obtain an alignment. Alignment takes your experimental tissue sections and helps align them to the Allen Brain Atlas CCFv3. A predictor network will evaluate your tissue and suggest the best fitting sections. Afterwards you will have to fine tune these predictions to best match your tissue.

Input Path = ~/belljar-sample/DAPI mages (or your experimental DAPI images) Output Path = ~/belljar-sample/Alignment

The Alignment method you choose depends on how you processed your tissue. The sample data is ‘single hemisphere’ so it should be set as such, if your experimental data is whole brain then choose ‘Both hemispheres’ option.


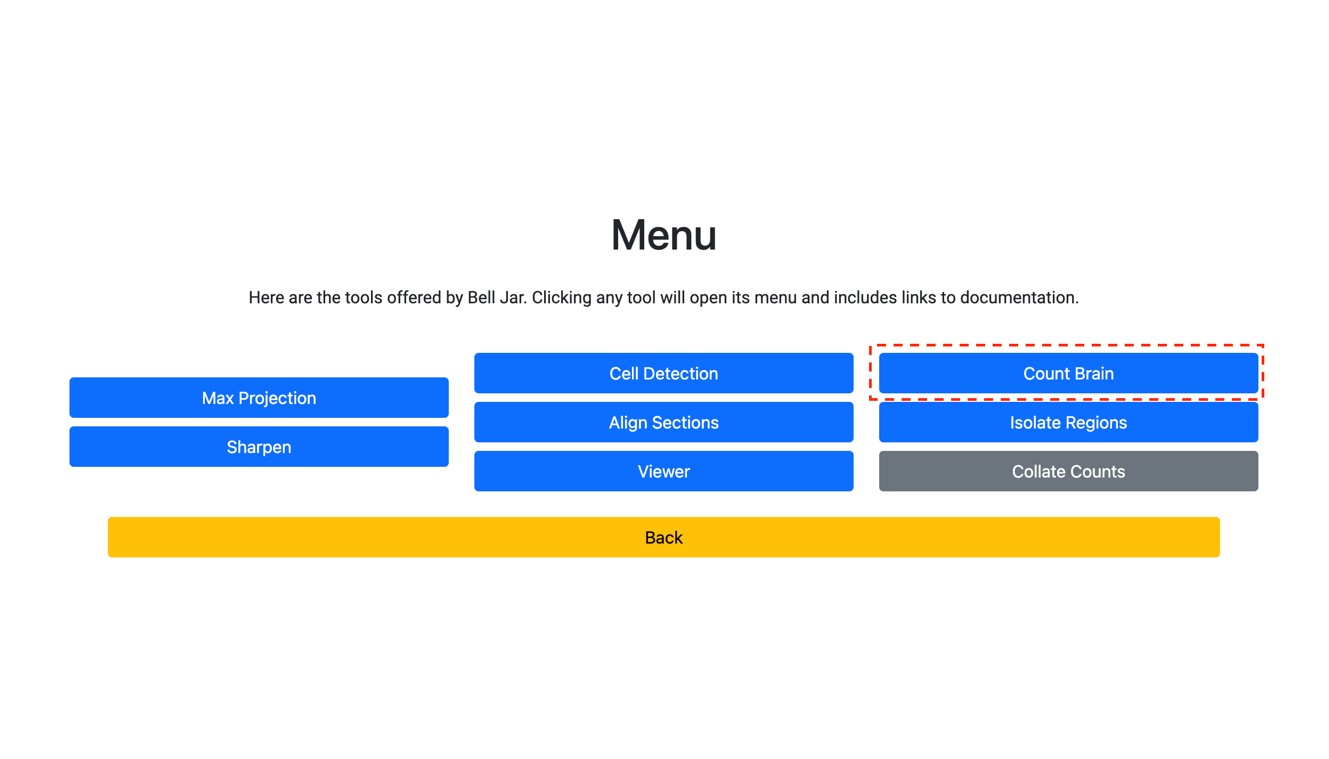


1. Finally, run the ‘Count Brain’ routine to obtain cell counts by region for the brain.

Predictions Path = belljar-sample/Detection

Annotations Path = belljar-sample/Alignment or belljar-sample/Adjustment if you made any adjustment

Output Path = belljar-sample/Counts

Feel free to compare counts against the bounding box and map images of the sample detections and alignment. count_results.csv will be found in the ‘Counts’ folder, thus Output path.

# 5. Troubleshooting common issues

# 5a. Alignment

- Error: "Index out of range … “, this indicates a mismatch between the number of images present when the alignment process began versus the actual output, ensure that once you start the process you are not modifying data until it has been completed
- Long Wait Times: If you experience a unusally long time for an alignment to complete, e.g. greater than 5 minutes for a single image, ensure you check the size of your images. Remember, for alignment **NO MORE than 2MB** in size for any given image.
- Frozen: The Napari window will appear to “freeze” once alignment begins to prevent modification once the process has started. If you are concerned the process may have crashed check the number of python processes running in your OS equivalent to “Task Manager”.

5b. Detection

- Error: "Index out of range … “, this indicates a mismatch between the number of alignment files provided, versus the number of signal files. Please ensure that you align every image you want to run detection on and that the number of aligned images matches the number of images passed in for detection
- Long Wait Times: If you experience an unusually long time for an alignment to complete, e.g., greater than 1 hour for a single image, ensure you check the size of your images. Expect large images (>1GB) to take a long time to process even with a single pass object detection.

5c. Adjustment

- Error: "Index out of range … “, this indicates a mismatch between the number of images and alignments provided to the adjustment tool. Ensure the right files and with no duplicates, or extraneous images are being passed to this function.
